# DNAJB12 and Hsp70 Mediate Triage of Misfolded Membrane Proteins for Proteasomal versus Lysosomal Degradation

**DOI:** 10.1080/27694127.2022.2139335

**Published:** 2022-10-26

**Authors:** Andrew Kennedy, Douglas M. Cyr

**Affiliations:** Department of Cell Biology and Physiology, Marsico Lung Institute, School of Medicine, the University of North Carolina at Chapel Hill, Chapel Hill, NC 27599, USA

## Abstract

The endoplasmic reticulum (ER) fills the cell with a continuous network of sealed membrane tubules and sheets. The ER is subdivided into microdomains mediating one-third of total protein biosynthesis, oxidative protein folding, secretion, protein quality control, calcium signaling, marcoautophagy/autophagy, stress sensing, and apoptosis. Defects in ER-calcium homeostasis underlie several diseases. Damage to the ER by misfolded membrane proteins is suppressed by specific HSPA/Hsp70 and DNAJ/Hsp40 chaperone pairs that select intermediates for ubiquitination and ER-associated degradation (ERAD) via the proteasome. The ER-transmembrane Hsp40 chaperone DNAJB12 and HSPA/Hsp70 also target toxic intermediates of misfolded membrane proteins for ER-associated autophagy (ERAA). DNAJB12-HSPA/Hsp70 maintain membrane protein degradation intermediates in detergent-soluble and degradation-competent states. DNAJB12-HSPA/Hsp70 also interact with the autophagy initiation kinase ULK1 on ER tubules containing ERAD-resistant misfolded membrane proteins (ERAD-RMPs). Omegasomes are ER microdomains where the autophagosome precursor or phagophore (PG) forms. ER tubules loaded with ERAD-RMPs enter omegasomes where they are converted into ER-connected PG (ER-PG). The Atg8 (autophagy related 8)-family member GABARAP (GABA type A receptor-associated protein) facilitates transfer of ERAD-RMPs from ER-PGs to autolysosomes (AL) that dock transiently with omegasomes. This article describes a model for DNAJB12-HSPA/Hsp70 action during the conformation-dependent triage in the ER of misfolded membrane proteins for folding versus proteasomal or AL degradation.

Biogenesis of transmembrane ion channels, G-protein coupled receptors (GPCRs), ATP binding cassette transporters (ABC-transporters), and P-type ATPases occurs in the ER. Folding inefficiencies in wild-type and disease-related mutant membrane proteins challenge the ER with the accumulation of potentially toxic misfolded intermediates. Retinitis pigmentosa is largely due to missense mutations in RHO (rhodopsin), a GPCR, that cause its misfolding, retention in the ER, and premature degradation. RHO contains a conserved disulfide whose formation is required for binding of the light sensing cofactor 9-cis-Retinal. Misfolded RHO intermediates lacking a disulfide are clients of ERAD. RHO mutants that cause retinitis pigmentosa, such as RHO^P23H^, misfold with degradation intermediates stabilized via a disulfide. RHO^P23H^ is therefore difficult to extract from the ER and is ERAD-resistant [1]. Dominantly-toxic conformers of RHO^P23H^ cause retinal degeneration and blindness, possibly through disruption of ER-membrane integrity. ERAA is proposed to suppress the accumulation of toxic and ERAD-resistant RHO^P23H^ (Figure 1).

ERAA is a pathway for the degradation of misfolded membrane protein intermediates in energized cells. ERAA occurs in parallel to ERAD, and the triage of misfolded membrane proteins between these pathways is mediated by hybrid protein quality control complexes containing DNAJB12-HSPA/Hsp70 and ERAD or autophagy initiation machinery. DNAJB12-HSPA/Hsp70 complexes help fold and assemble subunits of ion channels. DNAJB12-HSPA/Hsp70 also targets misfolded membrane proteins for ubiquitination through interaction with ER-associated ubiquitin ligases. Kinetically-trapped DNAJB12-HSPA/Hsp70 clients that contain tertiary structure, but fail to complete folding, such as RHO^P23H^, are not cleared from the ER and remain associated with DNAJB12-HSPA/Hsp70. This leads to the association of DNAJB12-HSPA/Hsp70 with autophagy initiation machinery and entry of ERAD-RMPs into ERAA.

The knockdown of DNAJB12 inhibits entry of RHO^P23H^ intermediates into ERAA. The mechanism for DNAJB12 action in ERAA requires its interaction with HSPA/Hsp70 and involves at least two functions. First, DNAJB12-HSPA/Hsp70 maintain nascent membrane proteins in a detergent-soluble conformation. The downstream fate of individual DNAJB12-HSPA/Hsp70 clients being determined by their innate folding kinetics on a case-by-case basis. Second, RHO^P23H^ stimulates the association of DNAJB12-HSPA/Hsp70 with the ULK1 complex. DNAJB12-HSPA/Hsp70 may target ULK1-RB1CC1/FIP200 (RB1 inducible coiled-coil 1) to ER tubules containing RHO^P23H^ to facilitate their conversion into ER-PGs (Figure 1A).

In support of this model, spots of RB1CC1, the targeting subunit of the ULK1 complex, are painted on ER tubules containing RHO^P23H^ at locations where they pass through the walls of WIPI (WD repeat domain, phosphoinositide interacting)-decorated omegasomal rings. RHO^P23H^-containing ER tubules located within omegasomes are decorated with Atg8-family proteins, which is a feature of ER-PGs. RHO^P23H^ is concentrated from the ER tubular network into ER-PGs in a selective process that excludes forms of RHO^P23H^ that are mutated to block disulfide formation. The transmembrane ER-chaperone CANX (calnexin) as well as DNAJB12, which both bind RHO^P23H^ in the ER, are excluded from ER-PGs. The exclusion of DNAJB12 and CANX from ER-PGs is consistent with ERAA being a selective process.

Live-cell imaging of ER-PGs containing RHO^P23H^ reveals them to dynamically interact with WIPI-decorated omegasomal surfaces. Notably, ALs marked with LAMP1 (lysosomal associated membrane protein 1) appear to dynamically dock with omegasomes and could therefore contact ER-PGs containing RHO^P23H^. AL association with omegasomes lasts on average for 22s and varies from short (10 s) to longer (70 s) associations. During apparent docking it appears that RHO^P23H^ is transferred from ER-PGs to ALs because after departure ALs contain new ~100-nm patches of RHO^P23H^. AL association with omegasomes is suggested to stimulate the autophagic transfer of RHO^P23H^ from ER-PGs to ALs.

A knockdown screen for Atg8-family protein requirements in ERAA revealed a role for GABARAP in the transfer of RHO^P23H^ from ER-PGs to ALs. Knockdown of all three GABARAP subfamily members blocks the accumulation of RHO^P23H^ in ALs. Yet, knockdown of all three LC3 subfamily members does not significantly hinder autophagic degradation of RHO^P23H^. Interestingly, GABARAP knockdown does not hinder the entry of RHO^P23H^ into ER-PGs, nor the association of ALs with omegasomes. Instead, loss of GABARAP hinders the transfer of RHO^P23H^ from ER-PGs to ALs.

LC3 family members appear sufficient to support early steps in ERAA, and GABARAPs play a specific role in autophagic exit of RHO^P23H^ from the ER. The GABARAPs interact with ULK1 with higher affinity than the LC3s, are implicated in focal activation of ULK1, and facilitate autophagosome fusion with ALs. These data provide a road map to explore the unknown mechanism for GABARAP action in RHO^P23H^ transfer from ER-PGs to omegasome-associated ALs.

A family of reticulophagy receptors that mediate the selective lysosomal degradation of ER that is damaged by stress and protein aggregates help maintain ER-homeostasis. RHO^P23H^ does not aggregate in the ER-membrane due to the action of DNAJB12-HSPA/Hsp70. The individual knockdown of six different reticulophagy receptors and the simultaneous knockdown of all six does not block autophagic degradation of RHO^P23H^. In ERAA, DNAJB12-HSPA/Hsp70 can play a role analogous to reticulophagy receptors. DNAJB12-HSPA/Hsp70 can also function with a yet to be identified reticulophagy receptor that facilitates ERAA. Proteomic studies are being conducted to identify new components of the machinery that triages ERAD-RMPs for ERAA instead of ERAD.

## Figures and Tables

**Figure 1.**
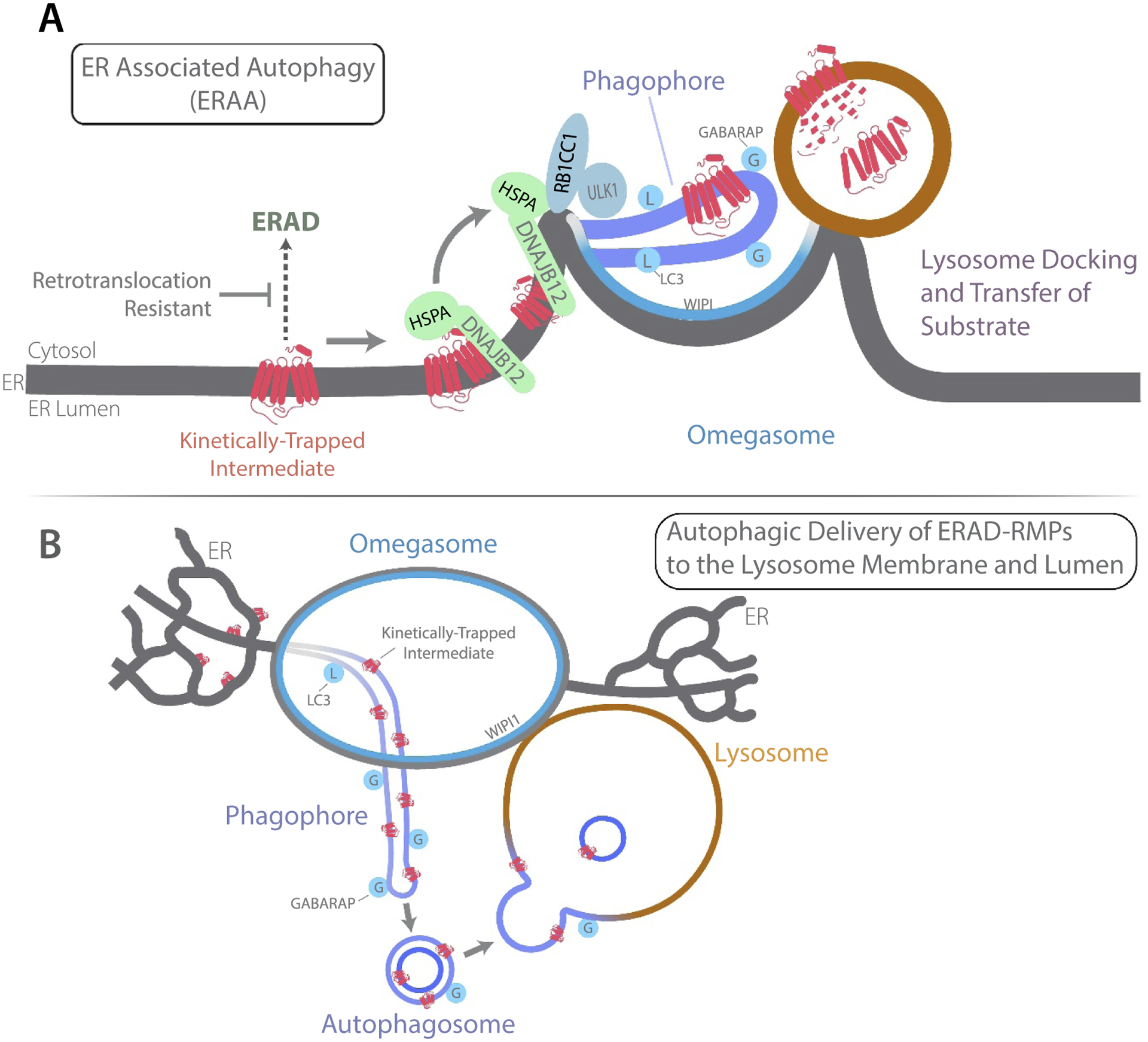
Degradation of misfolded membrane proteins by ER-associated autophagy. (**A**) Steps in the conformation-dependent triage of misfolded membrane proteins by ERAA. (**B**) Model for delivery of ERAD-RMPs from ER-PGs to the AL membrane and lumen. Abbreviations are defined in the main text.

## Abbreviations:

ABC-transporter: ATP binding cassette transporter
Atg8: autophagy related 8
DNAJB12: DnaJ heat shock protein family (Hsp40) member B12
ER: endoplasmic reticulum
ERAD: ER-associated proteasomal degradation
ERAA: ER-associated autophagy
ERAD-RMPs: ER-associated proteasomal degradation resistant membrane protein
GABARAP: GABA type A receptor-associated protein; GPCR, G protein-coupled receptor
HSPA/Hsp70: heat shock protein family A (Hsp70)
LAMP1: lysosomal associated membrane protein 1
MAP1LC3/LC3: microtubule associated protein 1 light chain 3
P-type ATPases: ion transporting ATPase
ULK1: unc-51 like autophagy activating kinase 1
RB1CC1/FIP200: RB1 inducible coiled-coil 1
WIPI: WD repeat domain, phosphoinositide interacting
